# A preliminary study of white matter disconnections underlying deficits in praxis in left hemisphere stroke patients

**DOI:** 10.1007/s00429-024-02814-3

**Published:** 2024-07-17

**Authors:** Elisabeth Rounis, Elinor Thompson, Michele Scandola, Victor Nozais, Gloria Pizzamiglio, Michel Thiebaut de Schotten, Valentina Pacella

**Affiliations:** 1 Chelsea and Westminster NHS Foundation Trust, London, UK; 2 Department of Brain Sciences, Imperial College London, London, UK; 3 MRC Cognition and Brain Sciences Unit, University of Cambridge, Cambridge, UK; 4 Department of Computer Science, UCL Centre for Medical Image Computing, University College London, London, UK; 5 Neuropsychology laboratory VR and Human Sciences Department, University of Verona, Verona, Italy; 6 Groupe d’Imagerie Neurofonctionelle, Institut des Maladies Neurodegeneratives-UMR 5293, CNRS CEA University of Bordeaux, Bordeaux, 33076 France; 7 Institute of Health and Wellbeing, University of Glasgow, Glasgow, UL UK; 8 Brain Connectivity and Behaviour Laboratory, Sorbonne Universities Paris, Paris, 75006 France; 9 IUSS Cognitive Neuroscience (ICON) Center, Scuola Universitaria Superiore IUSS, Pavia, 27100 Italy

**Keywords:** Limb apraxia, Meaningless gesture imitation, Splenium of the corpus callosum, Extrastriate visual areas, Disconnectome

## Abstract

Limb apraxia is a higher-order motor disorder often occurring post-stroke, which affects skilled actions. It is assessed through tasks involving gesture production or pantomime, recognition, meaningless gesture imitation, complex figure drawing, single and multi-object use. A two-system model for the organisation of actions hypothesizes distinct pathways mediating praxis deficits via conceptual, ‘indirect’, and perceptual ‘direct’ routes to action. Traditional lesion- symptom mapping techniques have failed to identify these distinct routes. We assessed 29 left hemisphere stroke patients to investigate white matter disconnections on deficits of praxis tasks from the Birmingham Cognitive Screening. White matter disconnection maps derived from patients’ structural T1 lesions were created using a diffusion-weighted healthy participant dataset acquired from the human connectome project (HCP). Initial group-level regression analyses revealed significant disconnection between occipital lobes via the splenium of the corpus callosum and involvement of the inferior longitudinal fasciculus in meaningless gesture imitation deficits. There was a trend of left fornix disconnection in gesture production deficits. Further, voxel-wise Bayesian Crawford single-case analyses performed on two patients with the most severe meaningless gesture imitation and meaningful gesture production deficits, respectively, confirmed distinct posterior interhemispheric disconnection, for the former, and disconnections between temporal and frontal areas via the fornix, rostrum of the corpus callosum and anterior cingulum, for the latter. Our results suggest distinct pathways associated with perceptual and conceptual deficits akin to ‘direct’ and ‘indirect’ action routes, with some patients displaying both. Larger studies are needed to validate and elaborate on these findings, advancing our understanding of limb apraxia.

**Supplementary Information:**

The online version contains supplementary material available at 10.1007/s00429-024-02814-3.

## Introduction

Limb apraxia, commonly referred to as ‘apraxia’, is a prevalent yet poorly understood disorder that often manifests after brain injury. Apraxia leads to the inability to execute voluntary skilled movements despite preserved elemental sensorimotor functions, coordination, and comprehension. It afflicts up to 40% of stroke patients, with unilateral lesions resulting in bilateral deficits in motor skills. Notably, patients with apraxia caused by left hemisphere stroke exhibit impairments in both their affected (contra-lesional) and unaffected (ipsi-lesional) hands (Heilman and Rothi 2003). Individuals affected by apraxia often struggle with everyday tasks like shaving, cooking or non-verbal communication through gestures. Consequently, apraxia is associated with a greater disability following a stroke, often necessitating increased reliance on caregivers for daily activities or placement in a nursing home (Donkervoort et al. 2006; Bickerton et al. 2012a).

**Fig. 1:**
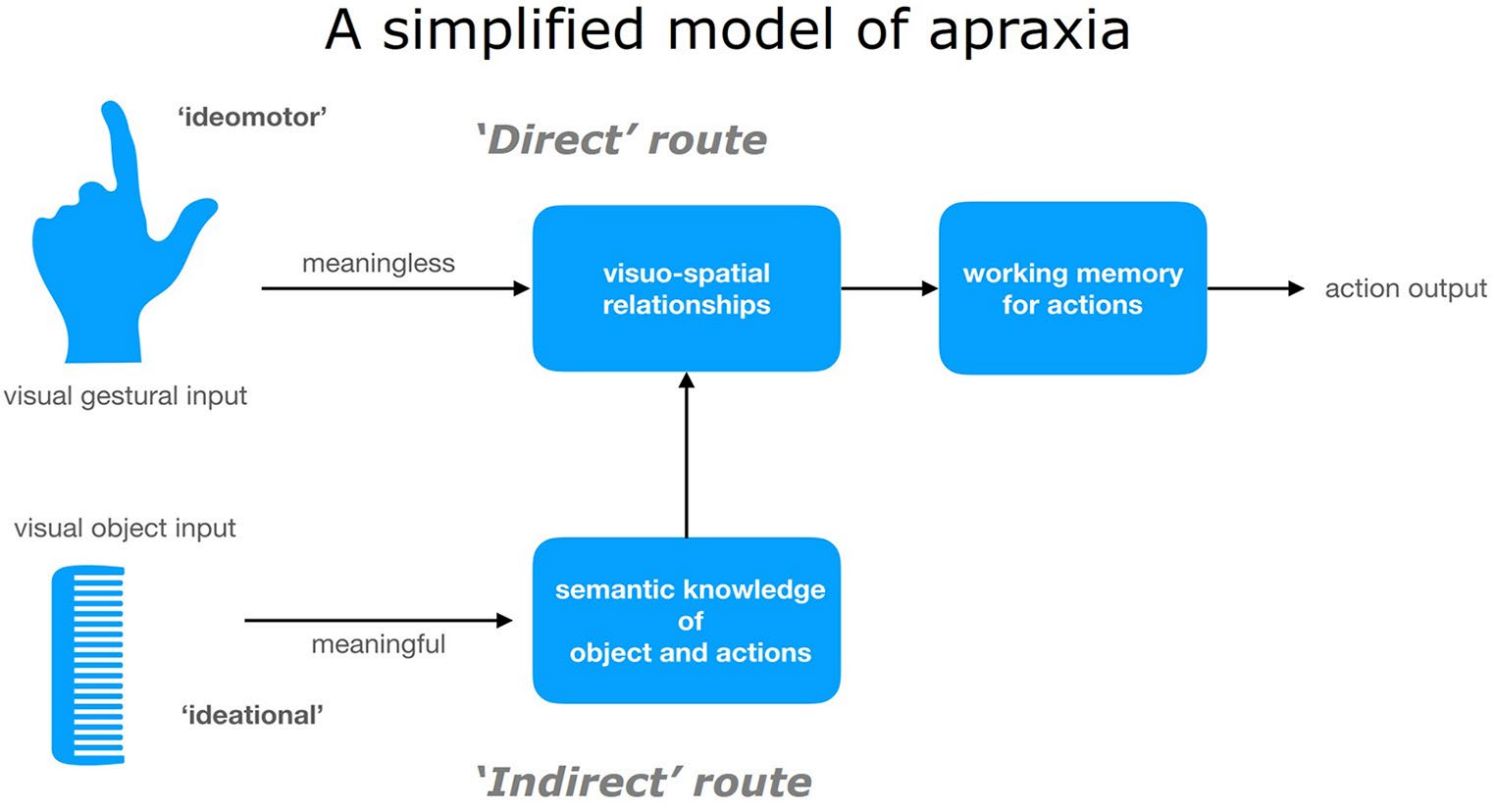
*The dual system model for action organisation* (Leiguarda and Marsden 2000), *adapted from* (Randerath 2023)

Traditional models of apraxia (as illustrated in Fig. 1) have historically differentiated between ‘conceptual’ and ‘production’ deficits based on errors observed in praxis tasks (Leiguarda and Marsden 2000; Canzano et al. 2016; Randerath 2023; Rounis and Binkofski 2023). In this framework, ‘ideational’ apraxia pertains to errors related to action planning or selection, while ‘ideomotor’ apraxia is associated with difficulties in executing skilful actions. ‘Ideational’ apraxia becomes evident through errors in tasks involving gesture ‘production’, such as pantomiming of or imitating familiar gestures (either transitive, e.g. showing how to use a toothbrush, or intransitive, e.g. showing how to wave goodbye), gesture recognition (transitive or intransitive), or multi-step actions (e.g. posting a letter). Conversely, ‘ideomotor’ apraxia is typically observed during the imitation of ‘meaningless’ gestures, often resulting in postural or spatiotemporal errors. The dissociation between ‘ideational’ and ‘ideomotor’ apraxia has led to the hypothesis that ‘ideational’ apraxia involves an ‘indirect’ route to action requiring retrieval of action sequences (or ‘movement formulae’) from memory or semantics. In contrast, ‘ideomotor’ apraxia was thought to follow a ‘direct’ route to action, relying primarily on visual analyses to replicate movements.

While behavioural distinctions between ‘ideational’ and ‘ideomotor’ subtypes of apraxia exist, there is little empirical support for distinct neuroanatomical substrates underlying the ‘direct’ and ‘indirect’ routes aforementioned. The initial hypothesis put forth by Hugo Liepmann in 1908 suggested a prominent role of the left parietal lobe in this disorder (Liepman, 1908). According to Liepmann, this brain region is crucial for storing, retrieving and implementing complex motor representations, which he termed ‘space-time plans of actions’ or movement ‘formulae’, particularly important to ideational apraxia. These movement formulae, more recently conceptualised as ‘blueprints’ (Hoeren et al. 2014) or even ‘affordances’ (Rounis and Humphreys 2015; Rounis and Binkofski 2023), are transmitted through ‘innervatory’ pathways to the primary motor areas for translation into actual movements (Leiguarda and Marsden 2000). Subsequent studies on apraxia proposed that it arises from ‘disconnections’ between parietal regions and fronto-striatal areas responsible for action execution (Geschwind 1965; Catani and ffytche 2005). This hypothesis gained support from the observation of apraxic deficits in patients who underwent callosotomies for intractable epilepsy (Gazzaniga et al. 1996). Additional case series involving patients with subcortical lesions impacting the basal ganglia, thalamus and adjacent white matter connections also reported significant praxis impairment (Pramstaller and Marsden 1996).

Recent investigations of apraxia have employed lesion-symptom mapping techniques to unveil the neural underpinnings of this complex condition. The ‘dual stream’ hypothesis, originally proposed by Goodale & Milner (Goodale and Milner 1992), has served as a fundamental framework for understanding praxis deficits, particularly in the context of the tasks mentioned earlier. This hypothesis postulates separate dorsal and ventral anatomical pathways, subserving action implementation and recognition processes. While this model continues to emphasise the crucial role of the left parietal cortex in apraxia (Goldenberg 2009), there is no evidence for separate neural pathways devoted to addressing ‘ideational’ and ‘ideomotor’ deficits. The dual stream hypothesis has provided a more nuanced account of deficits, suggesting that they may be more distributed in the brain (Binkofski and Buxbaum 2013; Rounis and Binkofski 2023). As a result, some investigators have questioned the validity of the ‘dual-route to action’ dichotomy, given that both ideational and ideomotor subtypes frequently co-occur in patients with apraxia (Buxbaum and Randerath 2018).

Studies of aphasia or neglect confront parallel challenges, in which sole reliance on lesion-symptom mapping may prove insufficient in elucidating the diverse spectrum of deficits observed in patients (Butler et al. 2014; Salvalaggio et al. 2020). The intricate interplay between brain structure and function, involving processes of ‘degeneracy’ and ‘diaschisis’, hinders the establishment of a one-to-one relationship between specific deficits and lesion locations (Price et al. 2010; Talozzi et al. 2023). Consequently, different brain lesions can cause the same functional impairment. A compelling approach to address these discrepancies has been identifying which white matter disconnections may lead to particular deficits (Pacella et al. 2019; Talozzi et al. 2023). However, until recently, it was difficult to derive white matter disconnections based on stroke lesion studies as the lesion often distorted white matter pathways going through it. New methods described by Foulon et al. (2018) have lately overcome this by applying patients’ lesion maps onto structural white matter connection maps from a large cohort of healthy volunteers from the human connectome project (HCP), scanned at high resolution. The aim is to comprehensively characterise various functional deficits resulting from disconnections between brain regions at the chronic post-stroke stages. A recent application of this ‘disconnectome’ approach not only mapped but also predicted several neuropsychological deficits from acute to chronic post-stroke stages (Talozzi et al. 2023).

In this study, we applied the same ‘disconnectome’ approach to explore which white matter disconnections characterise praxis abilities. The study, which was carried out during the ‘Neural 2023 hacking’ initiative, involved a small cohort of left hemisphere chronic stroke patients tested on detailed praxis tasks. This approach was felt to be promising in view of the fact apraxia has traditionally been regarded as a ‘disconnection’ syndrome (Catani & Ffytche 2005). However, only recently have neuroimaging techniques allowed the study of structural white matter disconnection in praxis disorder in tool use (Garcea et al. 2020) and pantomime (Rosenzopf et al. 2022) as well as in large cohorts of stroke patients with subcortical lesions (Schmidt et al. 2023). Due to the heterogeneity of the disorder, we entered continuous praxis scores of all patients in the analyses. Follow-up upvoxel-wise single-case analyses allowed a more detailed exploration of the anatomical underpinnings involving only those patients who had significant ideational and ideomotor deficits in gesture production and meaningless gesture imitation tasks, respectively.

## Methods

### Participants

This study involved 29 chronic left hemisphere stroke patients (M = 18, F = 11) with a first-ever stroke (24 had an ischaemic stroke, 5 had a haemorrhagic stroke). Their mean age was 59.5yo, range 29-79yo. All patients were tested and scanned within 12–62 months of stroke onset. Their mean years of education was 14.3 yrs (range 10–20 yrs). Exclusion criteria for the study included previous strokes, significant cognitive impairment precluding the ability to understand and provide written informed consent, or any other neurological or psychiatric condition. Table 1 outlines the demographic details of the patient population. Patients underwent a detailed and validated battery of apraxia tasks arising from the Birmingham Cognitive Study (Humphreys, 2012a, Bickerton et al., 2012b). All participants were right-handed (Oldfield 1971). Patients used their dominant hand or, if they had hemiparesis, their unaffected hand to complete the task, which is standard practice in praxis assessments (Heilman and Rothi 2003; Humphreys, 2012b, Bickerton et al., 2012a). A total of 14 patients used their right (dominant) hand, and 15 patients used their left (non-dominant) hand as shown under the column ‘Hand Used’ in Table 1.

Full written informed consent was obtained from all participants according to the declaration of Helsinki. The study was approved by the Health Research Authority, South Central – Berkshire Ethics Committee. Participants attended the Oxford Centre for Magnetic Resonance Imaging at the University of Oxford for neuropsychological testing and structural brain imaging.

**Table 1:** Demographic data

Patient Number	Lesion volume (1 mm^3^)	Months Post Stroke	Gender	Age	Years of Education	Hand Used
S1	320	12	M	66	20	R
S2	3042	12	M	68	11	R
S3	1198	16	F	78	13	R
S4	5585	24	F	50	12	R
S5	3384	18	M	54	12	R
S6	23,674	12	F	28	15	R
S7	12,879	36	M	62	17	R
S8	5375	24	F	55	19	R
S9	1117	24	M	60	16	L
S10	920	24	F	50	12	L
S11	4605	36	M	79	16	L
S12	14,052	24	M	51	12	L
S13	899	58	F	66	10	L
S14	197	28	M	58	17	R
S15	18,956	27	F	70	15	L
S16	15,291	12	M	68	15	R
S17	26,174	36	M	29	10	R
S18	28,382	24	M	44	17	L
S19	10,219	12	F	57	19	L
S20	9734	24	M	59	15	R
S21	12,290	36	M	35	15	L
S22	10,900	52	M	73	10	L
S23	4310	36	M	62	12	L
S24	3756	48	F	62	12	L
S25	8595	58	M	74	15	R
S26	3739	62	M	51	11	L
S27	2276	58	F	63	19	L
S28	700	62	M	77	16	L
S29	4253	12	F	75	14	R

### Neuropsychological assessment

This study used the battery of praxis tasks that was originally developed as part of the Birmingham Cognitive Screen (‘BCOS’) (Humphreys, 2012a). This screening comprises ‘gesture production’ tasks adapted from the Florida Apraxia screening tests (Power et al. 2010), and a ‘gesture imitation’ task from De Renzi’s meaningless gesture imitation screening for Apraxia (De Renzi et al. 1980). Whilst being faithful to original neurocognitive models of the disorder (De Renzi et al. 1980; Leiguarda and Marsden 2000), it has been adapted and validated for testing patients with cognitive impairments following a stroke, including patients with aphasia and neglect (Humphreys et al. 2012b, Bickerton et al. 2012a). The tasks patients were tested on are described in detail in previous publications (Bickerton et al. 2012b, Pizzamiglio et al. 2020, Rounis et al. 2021).

The praxis tasks included a meaningless gesture imitation task (involving imitation of meaningless finger and hand gestures to test ideomotor apraxia as in (Rounis et al. 2016); a gesture production task (requiring they pantomime three transitive, e.g. showing the use of a hammer, a glass and a salt cellar, and three intransitive, e.g. show me how to hitch-hike, military salute and stop), gesture recognition (recognising intransitive gestures – ‘come over’, ‘good’ and ‘goodbye’; as well as transitive gestures –such as the use of a cup, a key and a lighter), a single object-use task (in which patients were asked to demonstrate the use of a torch, a straw, a comb, a nail clipper, a screwdriver and matches with each object ‘in hand’), a multi-object use task (in which patients were asked to ‘light a torch’) and complex figure copy tasks (as in Pizzamiglio et al. 2020). The total scores for each praxis task were standardised to a maximum score of 100.

### Gesture scoring

Gestures for the Gesture Production, Meaningless Gesture Imitation, Single-Object use and Multi-Object use mentioned above were videotaped and later coded as correct or incorrect according to the scoring system detailed in Humphreys et al. (2012a), and Bickerton et al. (2012a). The scoring for each task was as follows: Gesture production, single and multi-object use were each scored out of 12, Meaningless gesture recognition out of 20: these tasks were scored on a Likert scale with a maximum score of 2 points for each gesture, 1 point if the gesture was conceptually correct and there were spatiotemporal errors and 0 points if the gesture was incorrect. Gesture recognition was scored for accuracy out of 6 (1 point for correct, 0 point for incorrect, for a total of 6 recognition items), and Complex Figure Copy out of 47 (see Humphreys et al. 2012b and Pizzamiglio et al. 2020 for scoring details).

Two independent coders scored the videos for each participant and each task. The final score for each task consisted of the average between the two scores. The average inter-coder reliability for all recorded tasks, defined by Cohen’s Kappa, was 0.76 for gesture production, 0.8 for meaningless gesture imitation, 0.76 for single and 0 0.75 for multi-object use tasks (together averaging = 0.77), demonstrating similar inter-rater reliability with previous studies (Buxbaum et al., 2005).

### Neuroimaging data acquisition

The patients had an MRI head scan using a Siemens 3 T Trio MRI scanner at the University of Oxford Centre for Clinical Magnetic Resonance research (OCMR) in a standard 12-channel head coil. High-resolution T1-weighted MR images were acquired using MP-RAGE sequence (repetition time 2040ms, echo time 4.7ms, field of view 174*192mm2; 192 slices, voxel size 1*1*1mm3, flip angle 8^o^ total scan acquisition time was 556 s. The imaging protocol included a Fluid Attenuated Inversion Recovery (FLAIR) scan (TR: 9s, TE 90ms, FOV 220 × 220 mm, axial plane slice thickness 3 mm, 47 slices). Imaging was acquired at the chronic stage on the same day as patients underwent neuropsychological testing.

### Stroke lesions

Individual T1 images in native space were used to manually delineate stroke lesions while consulting the FLAIR co-registered sequence, using MRIcron (https://www.nitrc.org/projects/ mricron). The lesions were identified and manually delineated on a slice-by-slice basis (GP). They were reviewed by a trained neurologist (ER) for accuracy in terms of lesion location and extent for each patient, providing cross-examination by two examiners, as standard practice in manual delineation (de Haan and Karnath 2018). Binary masks were made from the lesions using MRIcron (Rorden et al. 2012). They were subsequently normalised to the MNI 152 space (2 mm resolution) using the enantiomorphic normalisation tool in the BCB toolkit (http://toolkit.bcblan.com, Foulon et al. 2018).

### Neuropsychological white matter disconnectome

White matter disconnection maps were created by quantifying the pattern of white matter connections interrupted by each patient’s stroke lesion based on the high-resolution tractography of a healthy population from the Human Connectome Project (HCP) (Van Essen et al. 2013). The tractography dataset involved diffusion-weighted MRI data from 176 healthy participants, acquired at 7T. We derived white matter disconnections from stroke lesions, using the BCB toolkit according to the procedure described in Foulon et al. (2018).

We ran two types of analyses. First, generalised voxel-based linear regression models were implemented using a non-parametric ‘*randomise’ function implementedin* FSL (Winkler et al. 2014) to characterise deficits in each of the praxis tasks. The linear regression analysis allowed the exploration of voxels associated with the variance in the behavioural scores entered as a continuous variable. Disconnection maps were entered into the models as dependent variables, z-score transformed praxis scores for each task as predictors and we included age and lesion size as nuisance variables, correcting for multiple comparisons with 5000 permutations. Then, we ran a voxel-wise single-case Bayesian Crawford analysis in Python 3.8, adapted from the ‘singcar’ package (Rittmo and McIntosh 2021; https://CRAN.R-project.org/package=singcar). We based the single case analysis on the behavioural results, in which two patients (namely S2 and S18) scored below the cut-off for meaningless gesture imitation and one for gesture production, respectively. This parallels the approach used by Metzgar et al. (2022), which sought to distinguish ‘direct’ and ‘indirect’ pathways to action based on patient errors made in imitation versus gesture production tasks. Bayesian hypothesis testing compared the disconnection probability between each of the patients displaying the below cut-off performance in the respective tasks and the other left hemisphere patients in the group for each voxel. The posterior distributions obtained from Markov Chain Monte Carlo simulations were utilized to compute voxel-wise p-values. FDR correction was applied to control for multiple comparisons.

### Data availability

The raw dataset imported in the BCBtoolkit software to calculate individual patient disconnectomes is available at https://www.humanconnectome.org (7 T diffusion data). The script of the single case analysis is available at https://github.com/vale-pak/single-case-neuroimg. Patients did not consent for their video recordings to be shared or available. Any other anonymised behavioural or imaging data requests should be made to Dr E Rounis.

## Results

### Neuropsychological results

**Fig. 2:**
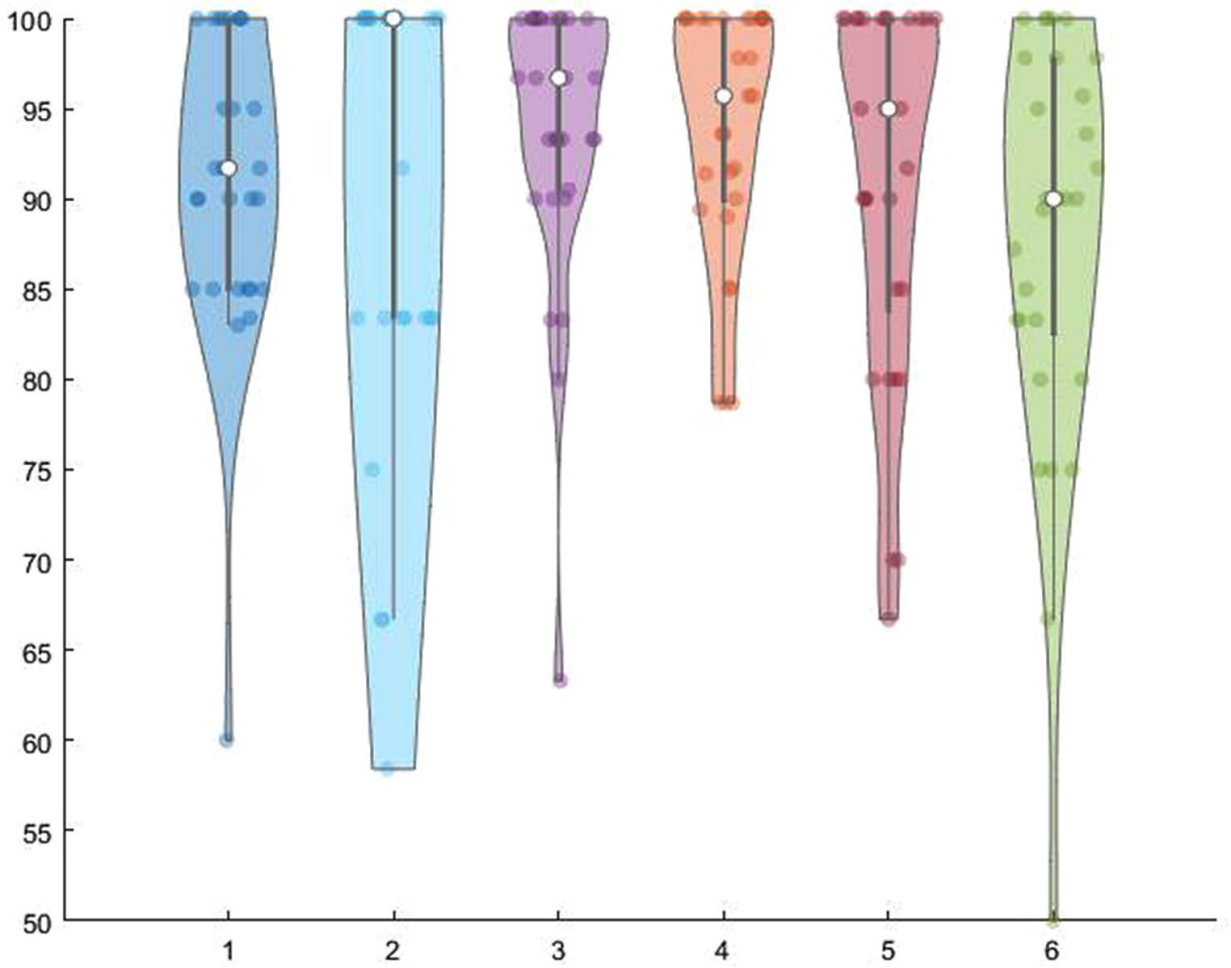
Violin plot representing patients’ performance on each of the tasks: The white dot represents the median for each individual task, and the black line represents the cut-off for all normalised scores (Bickerton et al. 2012b). The number of patients scoring below the cut-off for each task included: -meaningless gesture imitation (mean = 90.9, StDev = 8.7) 1 out of 29 (cut-off 75%). -gesture production (mean = 91.2, StDev = 12.3.) 2 out of 29 (cut-off 75%). -gesture recognition (mean = 93.7, StDev = 8.1) 2 out of 29 (cut-off 83%). -single-object use task (mean = 93.9, StDev = 7.2) 3 out of 29 (cut-off 85%). -multi-object use task (mean = 91.1, StDev = 10.6) 5 out of 29 (cut-off 85%). -complex figure copy (mean = 87.7, StDev = 11.7) 7 out of 29 (cut-off 83%)

The demographics and individual scores on the meaningless imitation task are shown in Table 2 (group level scores (in percent- %) for all tasks. These were as follows, with most patients’ performance at ‘ceiling’ (or above cut-off (either 75%, 83% or 85%, depending on the task, Fig. 2) based on BCOS normative data reported in the literature (Humphreys et al. 2012a).

**Table 2:** Patient performance in the apraxia tasks Behavioural results of patients in all remaining tasks in percent

Patient Number	Meaningless gestures (%)	Gesture Production (%)	Gesture Recognition %	Single Object use %	Multi Object use %	Complex Figure Copy%
S1	85	100	100	100	100	100
*S2*	***60***	***58.4***	***83.3***	***100***	***100***	***97.8***
S3	100	100	100	100	100	89.4
S4	90	100	100	100	100	93.6
S5	83	100	100	100	100	95.7
S6	95	83.4	100	100	100	87.2
S7	95	91.7	100	91.7	100	100
S8	100	100	100	100	100	97.8
S9	85	100	83.3	90	66.7	100
S10	90	100	100	100	100	100
S11	100	100	100	97.8	100	90
S12	90	83.4	96.7	100	85	80
S13	91.7	100	96.7	97.8	90	75
S14	85	100	63.3	85.1	80	83.3
S15	95	100	93.3	100	90	97.8
S16	100	75	96.7	100	90	85
S17	85	100	96.7	78.7	80	66.7
***S18***	***85***	***66.7***	***93.3***	***85***	***85***	***50***
S19	91.7	83.4	93.3	91.4	70	75
S20	100	100	93.3	89.4	95	100
S21	91.7	100	100	100	80	95
S22	85	83.4	100	93.6	80	91.7
S23	100	100	80	93.6	70	75
S24	90	100	90	78.7	95	83.3
S25	91.7	83.4	90.5	91.5	91.7	90
S26	90	100	90	78.7	95	83.3
S27	100	83.4	93.3	95.7	100	90
S28	100	83.4	93.3	95.7	100	90
S29	83.4	100	90	89	90	80

Patients S2 and S18 performed below cut-off scores for meaningless gesture imitation and geture production tasks. The single-case Crawford analyses for these respective tasks were therefore based on those patients

**Fig. 3:**
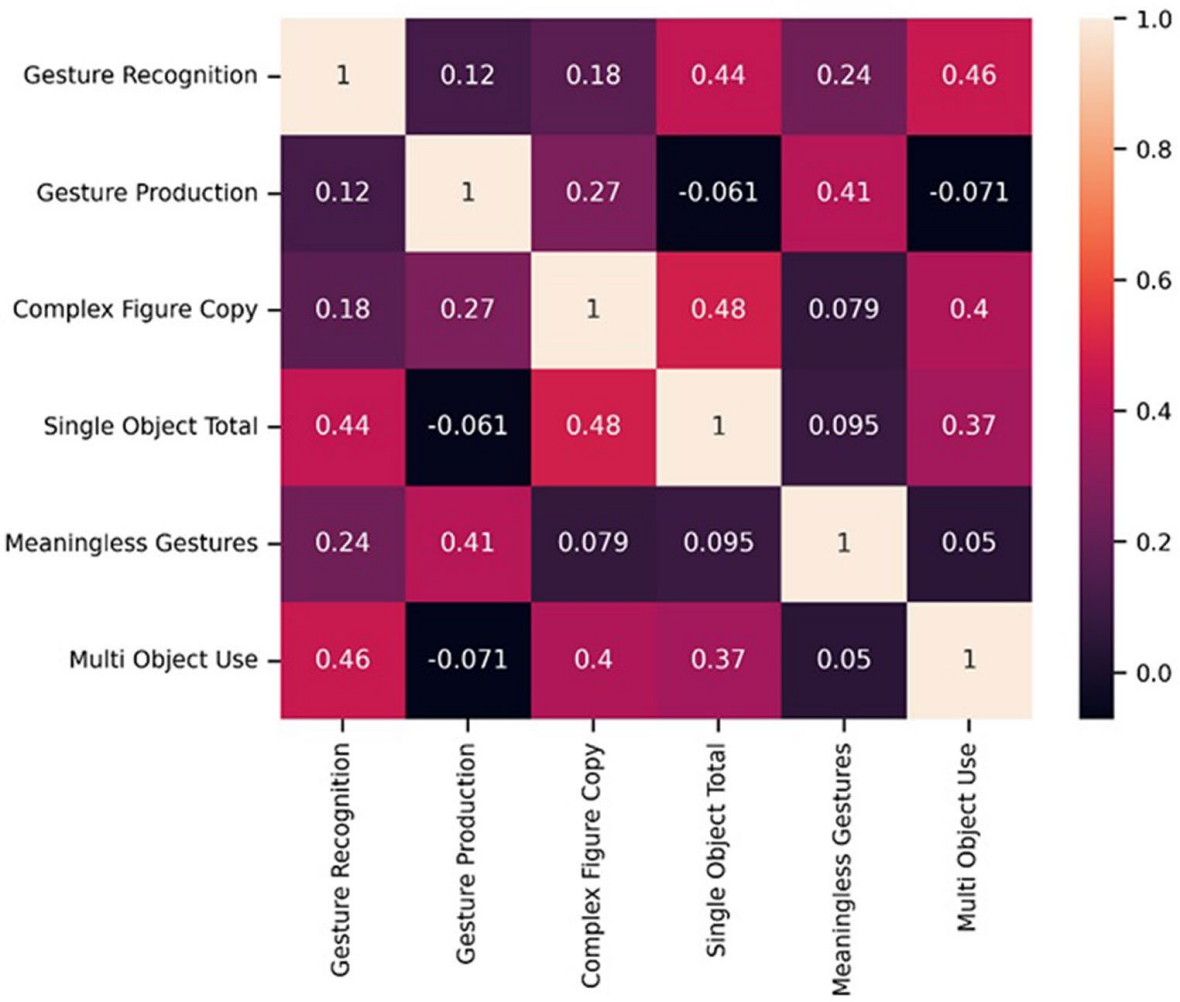
Correlation matrix of the patients’ performance on the task

A correlation matrix between the praxis assessment scores was computed and is shown in Fig. 3. Performance on several of the tasks was correlated: a strong correlation was found between the performance at the gesture recognition task and single object use (*r* = 0.44), and multi-object use (*r* = 0.46) tasks; between complex figure copy and single object use (*r* = 0.48), and multi-object use (*r* = 0.4) tasks. Moreover, the meaningless gestures task positively correlated with gesture production (*r* = 0.41).

Coding the accuracy of apraxia gestures has traditionally relied on Likert scale ratings (Heilman and Rothi 2003). Though there may be a lack of sensitivity compared to basing it on kinematic information, the latter is both impractical to apply in a clinical setting and remains insufficient as it may miss error information relating to posture inaccuracies (Goldenberg et al. 1996). Hence performance of patients on praxis tasks is often insensitive because they either perform at ceiling or floor. As a result, relying on dichotomised behavioural data (i.e. with vs. without apraxia) instead of considering the scores as continuous data often leads to a loss of power and reduced effect sizes (Cohen 1968). To mitigate against these limitations with praxis assessments, several lesion-symptom mapping studies (Manuel et al. 2013; Buxbaum et al. 2014; Hoeren et al. 2014) and the current ‘disconnectome’ one have entered patient scores as continuous rather than categorical variables, to allow for more sensitive neuroimaging group-levelanalyses.

### Imaging results

**Fig. 4:**
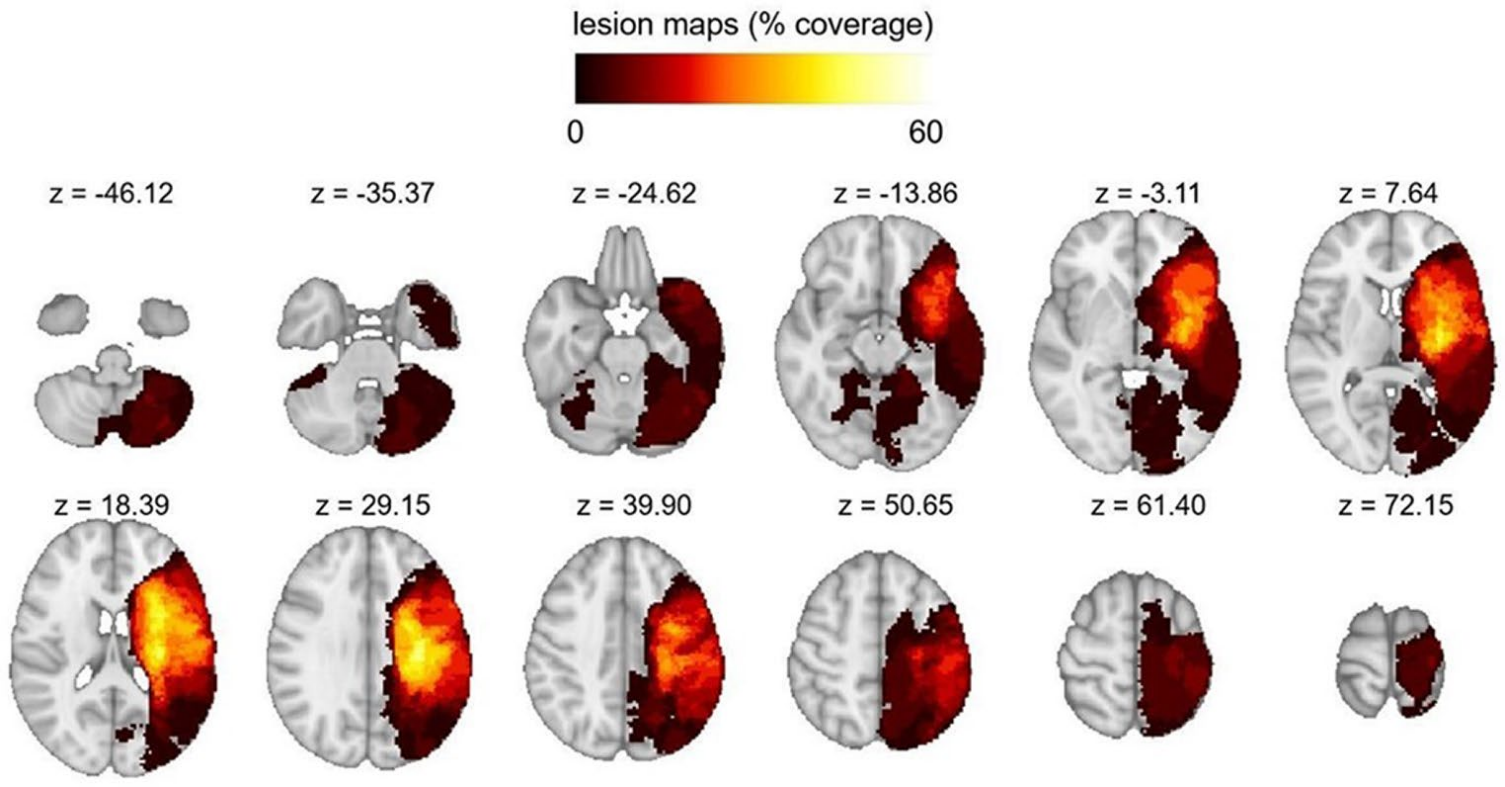
Lesion overlay map (in radiological convention, Right = Left side)

The lesion overlay map for the 29 patients included is shown in Fig. 3, displaying the lesions’ centre of mass in correspondence of (x=-36,y=-18,z = 16) (Fig. 4).

**Fig. 5:**
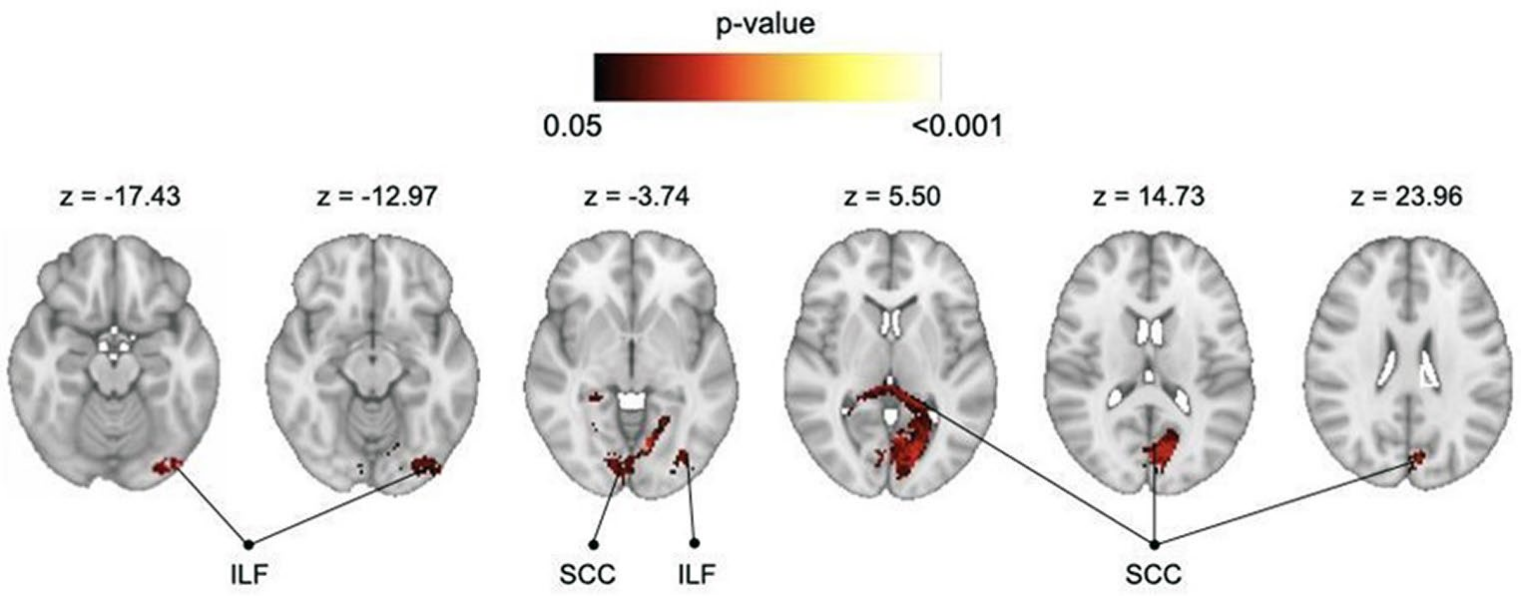
Disconnections associated with the meaningless gesture imitation task (in radiological convention, Right = Left side) thresholded at *p* < 0.05. Results show intra-hemispheric and inter-hemispheric disconnections via the inferior longitudinal fasciculus and the splenium of the corpus callosum. ILF: inferior longitudinal fasciculus; SCC: splenium of the corpus callosum

The only significant result from our group-level linear regression analyses is related to the meaningless gesture imitation task. This task revealed significant disconnection involving the splenium of the corpus callosum, extending to the inferior longitudinal fasciculus (Fig. 5). Although we did not identify any significant clusters for other tasks (gesture recognition, single and multiple object use, and complex figure copy tasks), there was a trend in the gesture production task, which was approaching significance, involving disconnection of the left fornix, reported at *p* < 0.1 uncorrected in Supplementary Fig. 1.

**Fig. 6:**
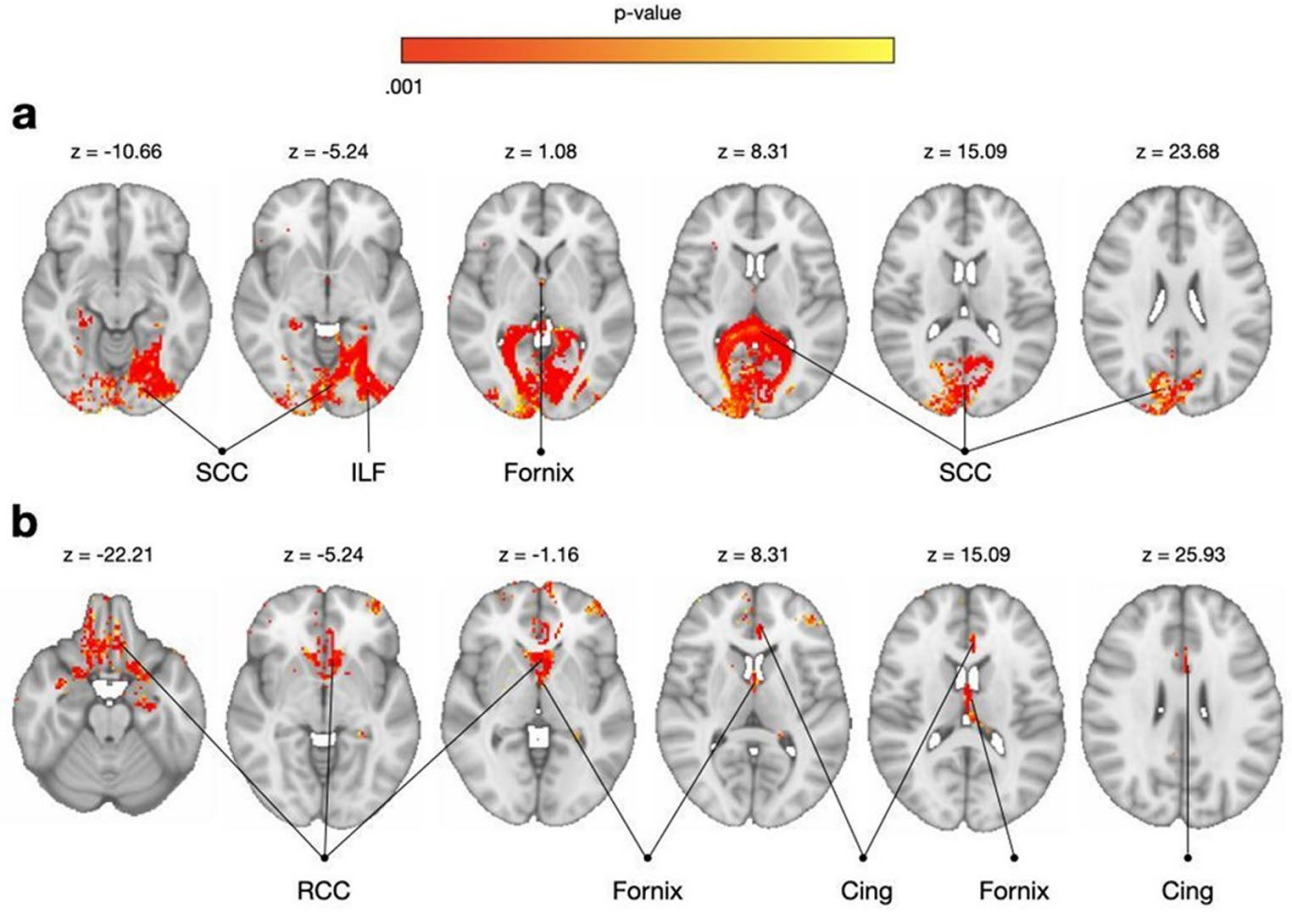
Single-case Crawford analysis on (**a**) the patient performing below cut-off in the meaningless imitation task and (**b**) the patient showing deficits in the gesture production performance. Cing: cingulum; ILF: inferior longitudinal fasciculus; RCC: rostrum of the corpus callosum; SCC: splenium of the corpus callosum; (in radiological convention, Right = Left)

A single-case Crawford analysis was performed comparing the patients in our cohort who fulfilled the criteria for limb apraxia on the meaningless gesture imitation task (S2) and the others. The same was done for another single patient who performed below cut-off on the gesture production task (S18), respectively (highlighted in Table 2). The single case analysis on the meaningless gesture imitation patient showed significant interhemispheric and intrahemispheric disconnections of the occipital lobes via the splenium of the corpus callosum and the left inferior longitudinal fasciculus (Fig. 6a). The single case analysis on the gesture production patient revealed the involvement of orbitofrontal cortices via the disconnection of the rostrum of the corpus callosum, and the limbic system via the disconnection of the fornix and the anterior cingulum (Fig. 6b).

## Discussion

In our preliminary study, conducted during ‘Neural 2023’ as part of the Brainhack initiative, we explored the theory that different networks underpin conceptual versus production-related deficits in praxis. This distinction is a cornerstone of traditional cognitive and behavioural models of apraxia (Leiguarda and Marsden 2000; Heilman and Rothi 2003; Rounis and Binkofski 2023). The ‘indirect’ route to action utilises familiar gestures by connecting the present visual scene with the gesture’s semantic memory. In contrast, a ‘direct’ route allows gesture execution based solely on visual transformation, sidestepping semantic knowledge. By probing the effects of lesion location on white matter disconnection pathways, our study was able to identify disconnections specific to the meaningless gesture imitation task. Our findings underscore the importance of the splenium of the corpus callosum in mediating ‘direct’ route to action. Due to the limited number of patients in our study, we observed only a trend for the ‘indirect’ route, suggesting a potential implication of the left fornix (Supplemental Fig. 1). We first discuss our group-level results then hone in on the single–case analysis findings before elaborating on methodological implications for future studies in apraxia.

### Mapping Praxis deficits in patients based on neuropsychological measures of apraxia

#### Group level analyses

In our study, we first predicted the disconnection pattern associated with praxis abilities in a group-level regression analysis. The z-score adjusted praxis scores for each patient were entered as continuous variables, a method now commonly applied in lesion-symptom mapping studies. By entering all the patients’ data in the analyses instead of dichotomising the scores into normal versus impaired in this analysis, we were able to take into account the severity of praxis impairment, thus maximising the statistical power of the analyses (Cohen 1968). This analysis identified a disconnection of the left hemisphere’s extrastriate visual areas *inter-*hemispherically through the splenium of the corpus callosum in patients exhibiting deficits in meaningless gesture imitation tasks.

This finding suggests a pivotal role of transcallosal pathways from the right hemisphere in meaningless imitation deficits underlying limb apraxia, thus challenging historical theories which purport apraxia primarily as a ‘left hemisphere’ syndrome (Liepman, 1908, Leiguarda and Marsden 2000; Pizzamiglio et al. 2020). Recent subdivisions of the dual stream hypothesis, particularly the “dorso-dorsal” subdivision within the dorsal stream, could offer anatomical support for our findings. This stream is suggested to facilitate sensorimotor transformations bilaterally, providing a “direct” route for action necessary for meaningless gesture imitation (Kalenine et al. 2010; Buxbaum et al. 2014). Furthermore, recent evidence suggests the predominant involvement of the right hemisphere in meaningless gesture imitation deficits, particularly in patients with neglect (Dressing et al. 2020). The role of the right hemisphere in imitation is further underscored by fMRI studies, which have implicated distributed networks spanning both hemispheres in this task (Iacoboni et al. 1999; Mengotti et al. 2012). Furthermore, intrahemispheric disconnection between extrastriate visual areas and frontal regions via the inferior longitudinal fasciculus (also identified in our group analysis) could disrupt the integration of this perceptual information into action plans (Watson et al., 2015; Zhang et al. 2021; Rounis and Binkofski 2023). Collectively, our results align with recent research emphasizing the importance of temporal lobe areas in integrating high-order perceptual gesture plans into sensorimotor transformations within each hemisphere (Buxbaum et al. 2014; Pizzamiglio et al. 2020; Sperber et al. 2019). The temporal lobes have also been implicated in several neurodegenerative disorders in which patients often have deficits in praxis, including Alzheimer’s disease and fronto-temporal dementias (Crutch et al. 2007; Bozeat et al. 2002).

Previous investigations have underscored the role of the opposite hemisphere in the recovery of visuo-perceptual abilities post-stroke and its influence on motor recovery (Karolis et al. 2019; Mattos et al. 2021). It is hypothesized to mediate “blindsight” following stroke, where individuals respond to visual stimuli despite lacking conscious awareness of them (Ajina and Bridge 2016; Celeghin et al. 2017; Danckert et al. 2021). Both blindsight and apraxia may arise from parallel intra or inter-hemispheric pathways. These pathways, which are typically redundant under normal conditions, may become relevant during disease states, contributing to either the exacerbation or amelioration of symptoms. For example, in blindsight, the presence of alternative visual pathways that bypass the primary visual cortex allows participants to accurately respond to visual stimuli despite lacking conscious awareness. Conversely, in the case of apraxia, disruptions in visual pathways may lead to deficits in motor planning and execution despite intact motor execution networks. These parallel conditions underscore the complex interplay between visual processing and motor function in both blindsight and apraxia, which may have implications for understanding the underlying mechanisms and potential treatment approaches. These findings complement dynamic changes identified in fMRI studies, which suggest that various forms of structural disconnection may cause differences in functional disconnections. As a result visual representations coming from extrastriate, ventral temporal areas may compete within frontal areas affecting the selection between different action goals (Gallivan et al. 2013; Zhang et al. 2021).

Our group-level analyses also revealed a noteworthy but non-significant trend for disconnection involving the left arm of the fornix in the context of a gesture production task. This lack of significance might stem from the small sample size of our study (29 patients), though we were able to see significant changes in the single-case analyses. Taken together, if these findings are corroborated in future, larger- scale studies, they could offer some scientific insight. Specifically they would concur with a previous conjecture that deficits underlying ‘ideational’ apraxia might arise from patients’ difficulties in retrieving actions ‘from memory’(Leiguarda and Marsden 2000). Thus a potential ‘indirect’ pathway to action could be mediated through the fornix, resulting in a disconnection of visual input from the hippocampus— a crucial area underpinning flexible cognitive behaviours within the limbic lobe (Rolls 2019; Mahon and Almeida 2024).

### Single case analyses

We followed up the group-level analyses with a single-case Crawford analysis, to help identify the white matter pathways associated with deficits in the most impaired patients, comparing them to the less affected patients in the meaningless imitation and gesture production tasks, respectively. The single-case statistical approach in the patient who performed poorly (below cut-off) on the meaningless gesture imitation task showed that his deficits were associated with disconnection of the splenium of the corpus callosum and inferior longitudinal fasciculus, confirming the group-level results. Of note, this patient was also impaired in pantomime tasks, reflecting the correlation found between meaningless gesture and gesture production tasks and supporting previous reports of difficulties dichotomising deficits pertaining to ideational versus ideomotor apraxia (Buxbaum and Randerath 2018). Nevertheless, single case analysis was conducted in the patient with the greatest deficit in the gesture production task who did not have a significant deficit in meaningless gesture imitation. This analysis identified disconnections involving the fornix, rostrum of the corpus callosum and anterior cingulum. These areas have been implicated in the storage and retrieval of action memories (Rolls 2019).

A study by Metzgar et al. (2022) used a comparable measure of brain disconnection. Their focus was on discerning which cluster of brain areas were disconnected, as opposed to our emphasis on white matter pathways. Out of a cohort of 29 left hemisphere stroke patients, they identified two individuals with small lesions who exhibited pronounced deficitsin pantomime of object use (which would represent a subset of our gesture production measure as it only included transitive gestures) versus meaningless gesture imitation tasks, respectively. Pantomiming familiar tool use correlated with disconnections between left temporal and parietal regions. On the other hand, deficits in imitating meaningless gestures were linked with disconnection between the left inferior and superior parietal lobules, and the left middle and superior frontal gyri. In addition to differences in task, in which the authors sought to investigate patients with rare yet isolated deficits, their study also differed in the neuroimaging analysis approach. We identified white matter tracts using a deterministic approach, whereas the Metzgar et al. (2022) study utilized a probabilistic ‘shortest path tractography’ approach. This approach investigated cortico-cortical disconnection overlooking large white matter tract pathways. However, building upon their findings, our research offers insights into white matter tract disconnections. This unveiled specific dis-connectivity patterns corresponding to the two-system praxis model (Leiguarda & Marsden, 2000). Moreover, our study introduces, for the first time, single-case Bayesian Crawford-Howells statistics to lesion analysis, strengthening the reproducibility and reliability of the findings.

Single-case studies have been contributing extensively to the advancement of knowledge on uncommon (Dalla Barba et al. 2018; Pacella et al. 2021; Metzgar et al. 2022) and unique neuropsychological syndromes (Thiebaut de Schotten et al. 2015; Pacella et al. 2020), though the results may be limited in their generalisability. Thus our two analyses offered a complementary approach to investigating praxis deficits by considering apraxia on a spectrum using the group analysis, yet deepening the findings using the single-case statistical approach.

### Mapping behavioural deficits using disconnection approaches

Despite clear distinction in the errors patients make on apraxia tasks, the evidence of separate networks underlying the two ‘routes to action’ – namely ‘ideational’ and ‘ideomotor’ apraxia, has been lacking (Leiguarda and Marsden 2000) and has not matched the similarly termed ‘dual stream’ hypothesis used to describe visuomotor pathways (Goodale and Milner 1992). Reasons for not identifying differences include limitations in our imaging and behavioural approaches to studying apraxia. As mentioned in the introduction, conventional imaging methods have historically relied on identifying lesion locations to map clinical neuropsychological manifestations onto the brain. However, it is essential to recognise that disconnection and diaschisis do not exclusively affect the lesioned area but instead extend their effects across broader and more distant networks, encompassing both structurally and functionally connected regions. Although diffusion tractography studies have enabled the in vivo exploration of white matter connections, their application can encounter limitations due to technical challenges affecting interpretation and analyses. For instance, lesioned brains caused by injury or stroke may cause distortions, making probabilistic seed-based tractography difficult to interpret. One way to circumvent such issue has been to integrate lesions onto large cohorts such as those from the Human Connectome Project (HCP, https://www.humanconnectome.org/) acquired using high-resolution imaging and advanced deterministic tractography techniques (Foulon et al. 2018; Talozzi et al. 2023). This innovative approach allows for a deeper understanding, as the acquisition of large healthy control datasets, like the HCP, enables the development of templates that can now facilitate the estimation of lesion locations and disconnections in a more accurate and comprehensive manner.

Furthermore, several challenges hamper the examination of the neuropsychological consequences of stroke. Firstly, the correlation matrix between behavioural scores highlighted the fact that several task categories correlate and may therefore underlie common cognitive mechanisms. This has also been recognised in studies of aphasia and neglect, where limited test selection sometimes fails to assess the full spectrum of heterogeneous deficits underlying these neuropsychological disorders. It is demonstrated in our study with the finding of significant correlations between several of the praxis tasks shown in Fig. 3. To address these issues, a promising approach involves employing data-driven techniques like principal component analyses to unveil underlying, latent patterns (Lambon Ralph et al. 2003; Butler et al. 2014). Notably, a prior study delved into apraxic deficits using principal component analyses, focusing on a set of eight apraxia tasks (Rounis et al. 2021). This analysis unveiled three core components that contribute to the disorder, encompassing aspects like posture selection, semantic control and sequencing deficits. Further efforts to identify white matter disconnections based on these components, particularly if validated with larger sample sizes, hold the potential to provide even more comprehensive insight into the mechanisms underlying limb apraxias, in the future.

Second, the small sample size in this study is a limitation as few of the patients had significant enough deficits to be deemed ‘apraxic’ according to cut-off scores. Despite identifying significant disconnections for meaningless gestures, we failed to identify disconnections for the remaining tasks. Nevertheless the validity of our results (namely of a significant relationship between behavioural deficit of interest and white matter disconnection) was confirmed using single-case Crawford analyses. This corroborates the approach used in Metzgar et al. (2022). It is important for future research to replicate and extend the results reported in this study by investigating larger samples of patients as has been done for other neuropsychological assessments (Talozzi et al. 2023). However the lack of detailed testing for apraxia in stroke studies has been a limitation in achieving this goal. While some studies have sought to establish double dissociations to mitigate this limitation (Metzgar et al. 2022), it is important to note that various deficits may co-occur (Buxbaum and Randerath 2018). Thus, seeking to dissociate them may result in the absence of common cognitive mechanisms underlying them (Butler et al. 2014; Rounis et al. 2021). For instance, patients may exhibit deficits in both pantomiming tool use and meaningless gesture imitation because of an underlying deficit in body schema (Rounis et al. 2021). Besides the possibility that big lesions can impair both systems jointly, a co-occurrence attributed to shared cognitive processes that underlie these deficits, as mentioned above, either in a ‘domain-general’ sense, such as the visual interpretation of gesture locations, or in a more ‘domain-specific’ manner such as those related to tool use or manipulation, may be inadvertently missed.

Finally a further caveat of our study might include the fact that the disconnections identified were derived from patients at the chronic post-stroke stage. These disconnections may have already undergone some degree of reorganization, and may not be reflective of the disconnections caused by the original lesions, at the acute stage. This could be further tested by investigating changes between the two stages in future longitudinal studies.

In conclusion, our study addresses a long-standing challenge of differentiating between conceptual and production related deficits in apraxia, shedding light onto its neural pathways and challenging historical assumptions about hemispheric dominance in apraxia. Future research in largersamples combining data-driven behavioural analyses with advanced neuroimaging holds the promise to further clarify the intricate mechanisms underlying apraxia deficits.

## Electronic supplementary material

Below is the link to the electronic supplementary material.

Supplementary Material 1
