# Supplementary material for: A preliminary study of white matter disconnections underlying deficits in praxis in left hemisphere stroke patients
Source: Brain Struct Funct. 2024 Jul 17;229(9):2255–68. doi: 10.1007/s00429-024-02814-3 (PMC11611995; doi:10.1007/s00429-024-02814-3)

**Supplementary Material:**

Supplementary Figure 1: Disconnectome associated with gesture production (thresholded at P<0.1, min p=0.073), identifying fornix (WM pathway to hippocampus) in gesture production deficit. If confirmed in a larger patient cohort, this finding would support the suggestion of a deficits in retrieving gestures ‘from memory’ (Cubelli et al., 2000).


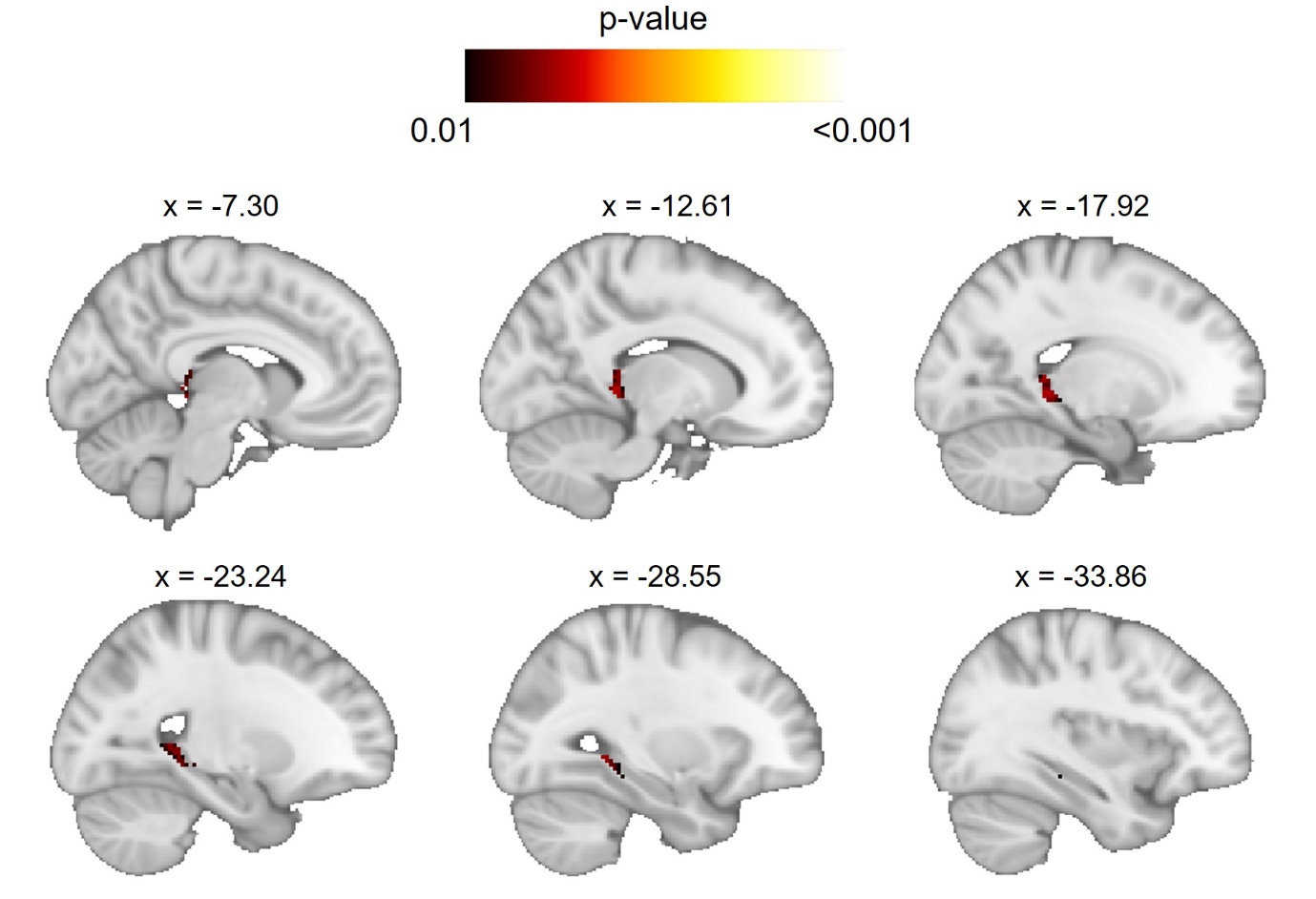

Supplement: Supplementary file 1 — Supplementary Material 1 [file 429_2024_2814_MOESM1_ESM.docx]
